# A simple hydrothermal route for the preparation of novel Na–Y–W nano-oxides and their application in dye degradation

**DOI:** 10.1039/d1ra07645k

**Published:** 2022-02-08

**Authors:** Saeed Moshtaghi, Masoud Hamadanian, Masoud Salavati-Niasari

**Affiliations:** Institute of Nano Science and Nano Technology, University of Kashan Kashan P.O. Box 87317-51167 Iran hamadani@kashanu.ac.ir salavati@kashanu.ac.ir +98 31 5555 29 30 +98 31 5591 2383

## Abstract

The fabricated NaY(WO_4_)_2_ was identified through diverse analysis methods. Therefore, to optimize NaY(WO_4_)_2_ morphology, saccharide carbohydrates were manipulated as a capping agent. In this study, glucose, fructose, lactose, cellulose, and starch were utilized as the capping agents. SEM images show that fructose was the optimal capping agent for achieving uniform and well-shaped nanoparticles. The photodegradation of organic dyes such as M.O and Rd.B by NaY(WO_4_)_2_ was evaluated under UV and Vis light. The bandgap energy of the as-prepared sample was measured by the Tauc plot, and was found to be nearly 3.85 eV. To study the photocatalytic characteristics, the influence of dye dosage and reusability on photodegradation behavior were investigated.

## Abstract 2

NaY(WO_4_)_2_ nanoparticles were fabricated *via* a simple hydrothermal method using saccharide carbohydrates as capping agents. The photocatalytic behavior of the as-prepared NaY(WO_4_)_2_ nanostructures was studied.

## Graphical abstract

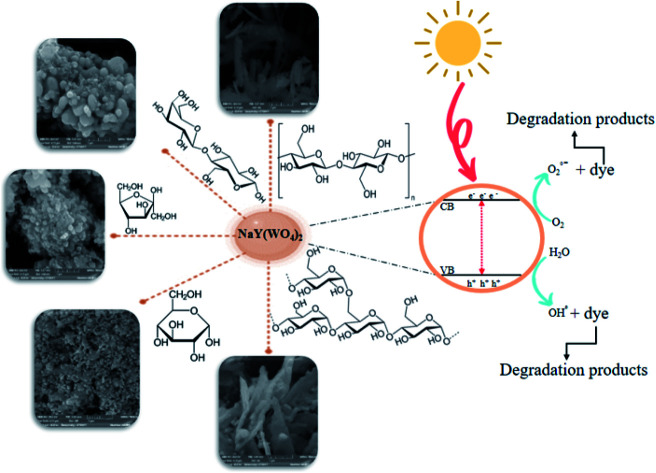

## 1. Introduction

Water contamination has turned into one of the recurrent universal ecosystem concerns worldwide currently.^1^ However, this is chiefly due to the development of expanding society, industrialization, and agriculture, which increase the contamination of pure and fresh water.^2^ One of the leading sources of water pollution is industrial dyes.^3,4^ These dyes contain non-biodegradable, highly toxic, and colored pigments that are harmful to living organisms.^5,6^ Thus, there is an urgent demand for some impressive and effective technologies and treatment methods.^7,8^

Nanotechnology is one of the top five technologies for wastewater treatment.^9–12^ It has attracted more attention in the large-scale range of applications because of the unique size-dependent characteristics such as high surface area, and catalytic, antimicrobial, photosensitive, optical, electrochemical, and magnetic properties.^13,14^

Among the nanostructures, the tungstate group is a substantial and particular nano-oxide.^15–19^ The momentous sub-group of the tungstate group is NaY(WO_4_)_2_, a double alkaline rare-earth tungstate that involves a large group of structures with formula MRE(WO_4_)_2_, in which M denotes a monovalent cation (Na^+^, K^+^), Re is a trivalent cation (Y^+3^, Yb^+3^), and WO_4_ is a divalent anion, which has various utilizations, such as scintillators,^20^ displays (LEDs),^21^ catalysis,^22^ optical waveguide,^23^ and environmental control.^24^

NaY(WO_4_)_2_ was manufactured through a solution-based method due to the superb individual chemical characteristics and morphology rather than the solid-state approach, which requires high temperature to obtain pure particles.^25^ The various synthesis methods applied in the synthesis of NaY(WO_4_)_2_ nanostructures include, solid-state,^26^ sol–gel,^27^ combustion,^28^ and hydrothermal.^29–31^ Among all the methods, the hydrothermal method has gained growing attention due to a range of advantages including broad area of materials, purity of products, uncomplicated usage, and short reaction time.^32–34^

In the study, sodium yttrium tungstate nanoparticles were fabricated by a hydrothermal approach, and then various saccharides, such as glucose, fructose, lactose, cellulose, and starch were applied as capping agents. The obtained particles were then heated at specified temperatures to produce highly active NaY(WO_4_)_2_ nanoparticles, which were utilized as photocatalysts. The role of different dyes, type of capping agent, and reusability of photocatalyst were also investigated. Ultimately, the influence of pollutant dosage change on the photodegradation performance in the presence of induced-light was also investigated.

## 2. Experimental

### 2.1 Materials and characterization

The chemicals used for the synthesis of NaY(WO_4_)_2_ nanoparticles including Na_2_WO_4_·2H_2_O (99%), Y(NO_3_)_3_·5H_2_O (99%), glucose, fructose, lactose, cellulose, and starch were purchased from Merck and Sigma-Aldrich Companies and used without further purification. Furthermore, deionized water was used as the solvent. XRD patterns were recorded through a diffractometer using Philips X-ray brand, utilizing filtered Cu Kα Ni irradiation. SEM images were taken on a LEO model 1455VP device. Prior to capture, the samples were covered with an Au thin film to render the sample surface conductive, block stacking and achieve good contradiction. The FT-IR spectra were recorded on an FTIR5000 series of Galaxy spectrophotometers. The electronic spectrum from the nanoparticles was obtained on a UV-Vis scanning spectroscope (Shimadzu, UV-2550).

### 2.2 Hydrothermal synthesis of sodium yttrium tungstate nanostructures

**Scheme 1:**
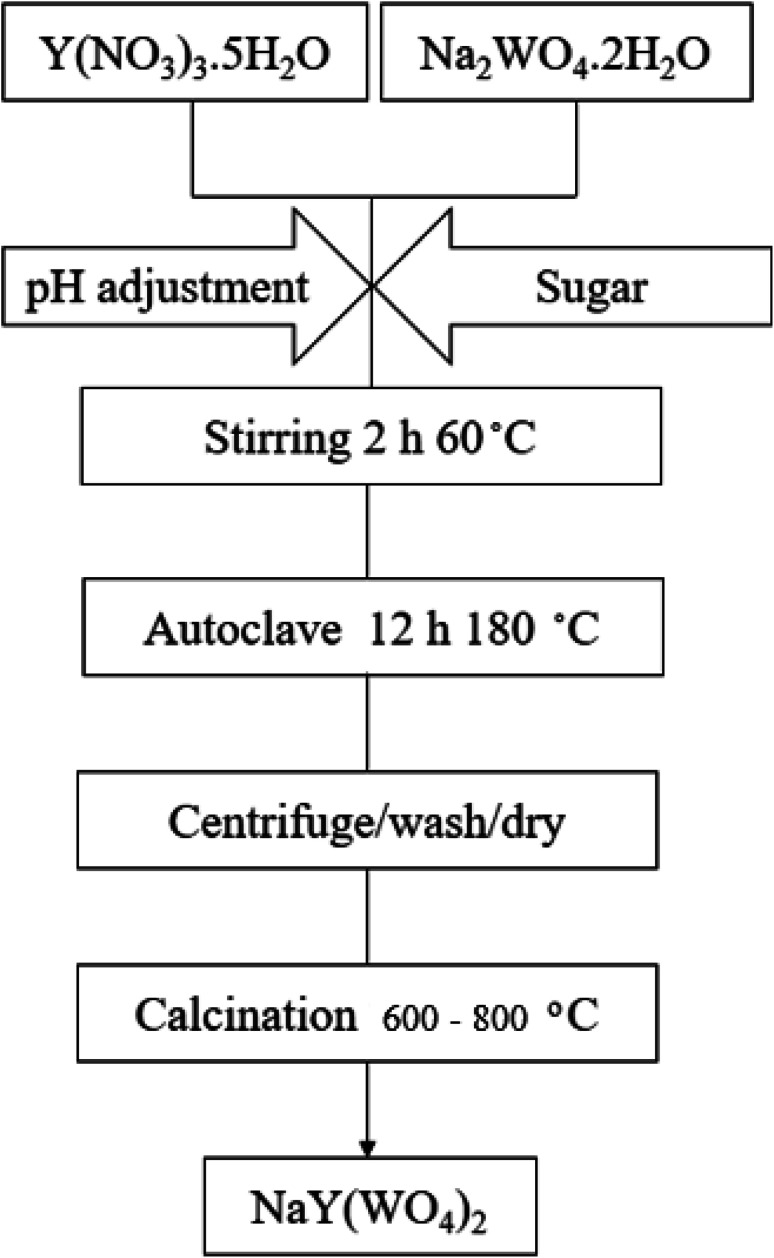
Schematic for NaY(WO_4_)_2_ synthesis using the precursors.

In this study, sodium yttrium tungstate nanoparticles were fabricated by the hydrothermal approach, utilizing Na_2_WO_4_·2H_2_O, Y(NO_3_)_3_·5H_2_O, and saccharide precursors. The flow diagram for the fabrication of NaY(WO_4_)_2_ nanoparticles is given in Scheme 1. According to the scheme, sodium precursor (0.1 mmol) and Y (0.1 mmol) were combined with the accurate volume of deionized water at 60 °C. Furthermore, an the aqueous solution of the saccharide agent (2.4 mmol) was added into the reaction media and agitated vigorously at 80 °C. Thereafter, a 1 M ammonia solution was used to adjust the pH of the solution to 10. The above-mentioned solution was placed in a beaker with a specific amount, and later placed in an autoclave, followed by heating at 180 °C for twelve hours. A dark brown product was obtained after centrifuging and rinsing three times. The precipitate was dried at 90 °C for one day and night in an oven. Finally, the precipitated dark brown powder was heated at 600–800 °C for three hours in an oxygen furnace. To show the various effective parameters analyzed on the NaY(WO_4_)_2_ nanostructures, the experimental conditions are tabulated in Table 1.

**Table 1:** Various experimental conditions for the synthesis of NaY(WO_4_)_2_

Sample No.	Calcination T.	Capping agent	pH adjustment	Ratio of capping agent to ion
S1	800 °C	Glucose	10	12 : 1
S2	800 °C	Fructose	10	12 : 1
S3	800 °C	Lactose	10	12 : 1
S4	800 °C	Cellulose	10	12 : 1
S5	800 °C	Starch	10	12 : 1

### 2.3 Dye degradation process

The degradation of organic dyes was accomplished in a black box setup comprising 200 mL of the dissolved dye in water solvent with high concentration (20 ppm) at pH = 3 for methyl orange and pH = 10 for rhodamine B, 0.005g L^−1^ of NaY(WO_4_)_2_ catalyst under Ultraviolet-Visible irradiation. The as-prepared dye solution including the photocatalyst was placed in dark for about 30 min under vigorous magnetic stirring at room temperature (25 °C). Afterward, the as-prepared system was placed near a UV or Vis lamp. The standard distance between the as-prepared suspension and the Vis and the UV lights was adjusted to 25 cm and 40 cm, respectively. A Xenon lamp (500 W) and an Osram lamp (400 W) were placed in a quartz vessel as the Visible and UV light irradiators, respectively.

**Fig. 1:**
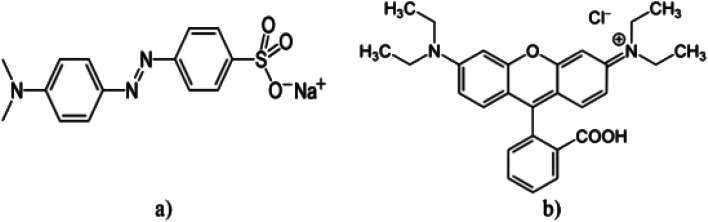
Chemical structure of (a) methyl orange (M.O) and (b) rhodamine B (Rd.B).

Thereafter, the specimens were cleaned and filtered, and the residual photocatalyst separated by centrifuging. Consequently, samples were tested using the UV-Vis spectrometer. The structure of dyes is shown in Fig. 1. The percentage of the photocatalytic degradation of aquatic contaminants was calculated as follows:1
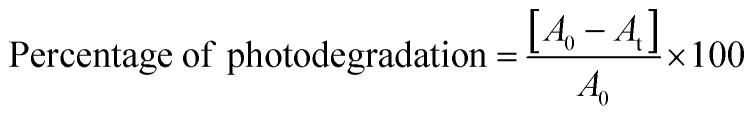
where *A*_0_ and *A*_t_ are the concentrations of contaminants before and after degradation, respectively (eqn (1)).^35–38^

## 3. Results and discussion

**Scheme 2:**
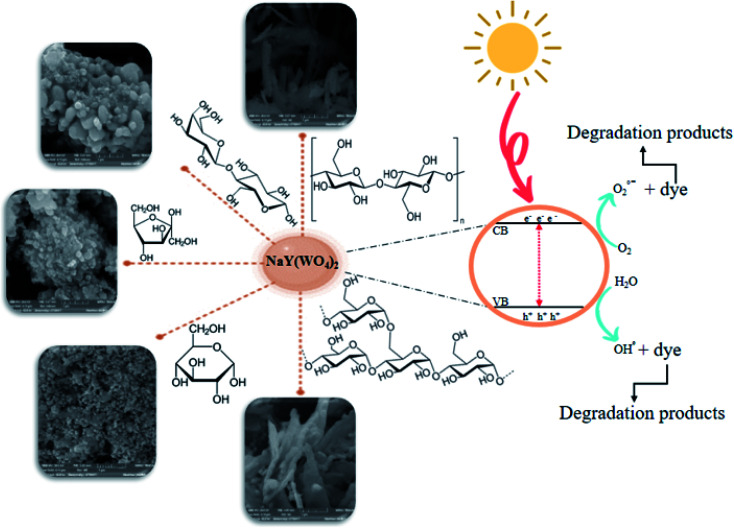
The graphical mechanism for the synthesis and photodegradation performance of NaY(WO_4_)_2_ nanoparticles.

**Scheme 3:**
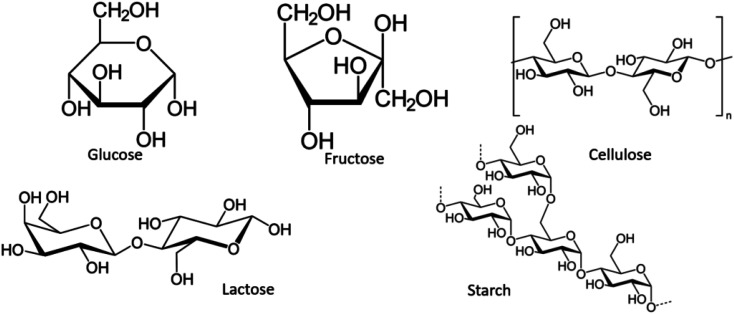
Chemical structure of disaccharide carbohydrates.

This article presents a sodium yttrium tungstate fabricated as a photocatalyst that is sensitive to UV light with a saccharide carbohydrate support as the capping agent (Scheme 2). The adjusted influence of NaY(WO_4_)_2_ nanoparticles was examined in determinative parameters, such as the change of saccharide purity, morphology, chemical, and physical properties. The main purpose of using saccharides is the steric effect and also the functional groups which prevent the aggregation of NaY(WO_4_)_2_ nanoparticles. The main functional groups of saccharides are OH and C=O, where the conjugating function of the mentioned functional groups with central metal ions leads to a spatial effect in the surroundings of the manufactured particles and also prevents aggregation.^39–41^ The structure of saccharides is shown in Scheme 3.

### 3.1 Morphology investigation

**Fig. 2:**
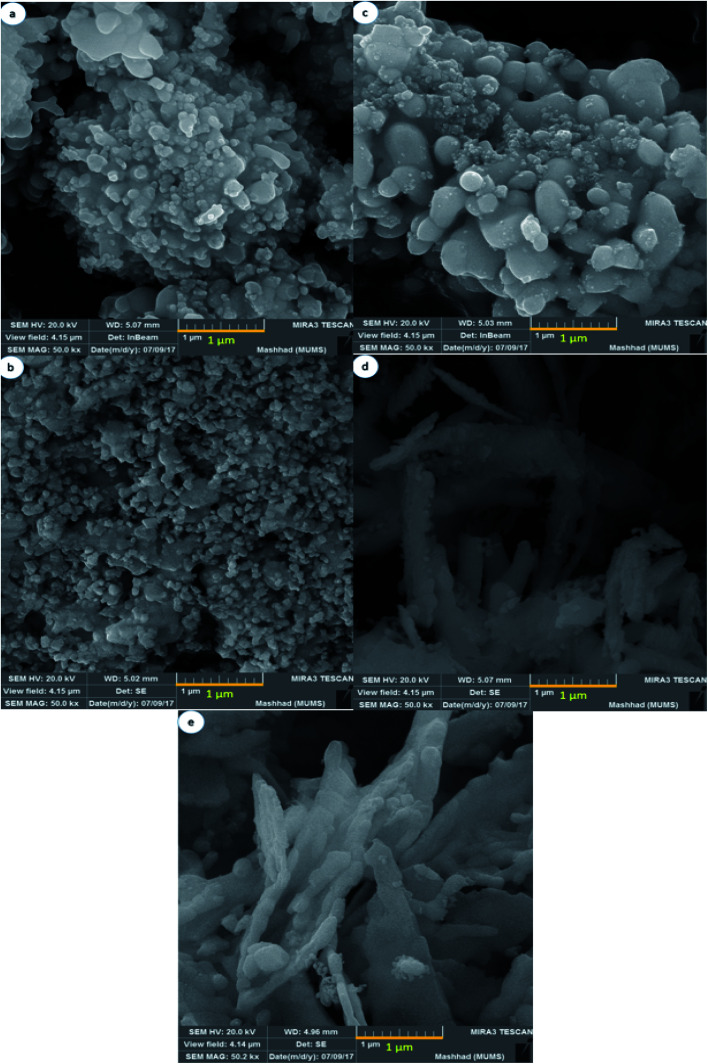
FE-SEM image of NaY(WO_4_)_2_ nanostructures fabricated by (a) glucose, (b) fructose, (c) lactose, (d) cellulose, and (e) starch as capping agents.

To study the effect of multiple saccharides on the shape and morphology of NaY(WO_4_)_2_ nanostructures, SEM images were obtained. Fig. 2a presents the SEM image of NaY(WO_4_)_2_ nanoparticles formed with glucose as the capping agent (sample S1). Glucose is an uncomplicated and the most abundant monosaccharide, which is also the most essential source of energy with the acyclic structure of aldohexose groups. Hence, due to the spatial disturbance of the glucose and nanoparticles, the nanoparticle morphology of as-synthesized NaY(WO_4_)_2_ was achieved, with an average particle size of nearly 102 nm.

Fig. 2b shows the uniform-shaped nanoparticles synthesized with fructose as a cyclic monosaccharide capping agent (sample S2). The cyclic structure of the fructose and two reactive CH_2_OH functional groups resulted in the uniform and superb NaY(WO_4_)_2_ nanoparticles. The average size of the above-mentioned nanoparticles was about 78.5 nm.

Lactose is a disaccharide carbohydrate comprising of glucose and galactose subunits. As indicated, NaY(WO_4_)_2_ microspheres were fabricated using lactose (sample S3). Excessive amount of the capping agent (lactose) led to aggregation; nevertheless, it was crucial to consolidate the concentration of electrolyte required to provoke a swift decrease in particle aggregation, and the reduction in particle size because of the slight electrostatic repulsion (see Fig. 2c).

As Fig. 2d demonstrates, the NaY(WO_4_)_2_ microfibers were synthesized utilizing cellulose as the linear polysaccharide. The structure of the cellulose restricted the growth of NaY(WO_4_)_2_ in a particular path. The tube-like morphology of the aggregated as-prepared nanostructure is due to the high temperature of the pre-solution of the mixture of precursors because of the low solubility of cellulose in water. The particles were about 96 nm on average. Fig. 2e shows the image of sample S5 microplates fabricated *via* a hydrothermal approach using starch in a 1 : 12 molar ratio to the central ion. The average size of the particles was 197 nm, approximately. Furthermore, the polysaccharide structure of the starch and glycosidic bonds caused the accumulation due to the high concentration of CH_2_OH in the reaction media.

As observed from the SEM images, the optimized morphology with the narrow size distribution and uniform morphology is the sample S2 fabricated by hydrothermal method using the optimized amount of fructose as the capping agent.

To achieve NaY(WO_4_)_2_ particles with favorable characteristics, the control and measurement of the size distribution is crucial.^42,43^ Determination of the size distribution can also be done by Dynamic Light Scattering (DLS) but requires additional analysis parameters.^44^ Nevertheless, it is difficult to measure the particle size accurately due to the adhesion of particles. Therefore the measurement of the particle size is accomplished by analyzing SEM images *via* Digimizer software.

**Fig. 3:**
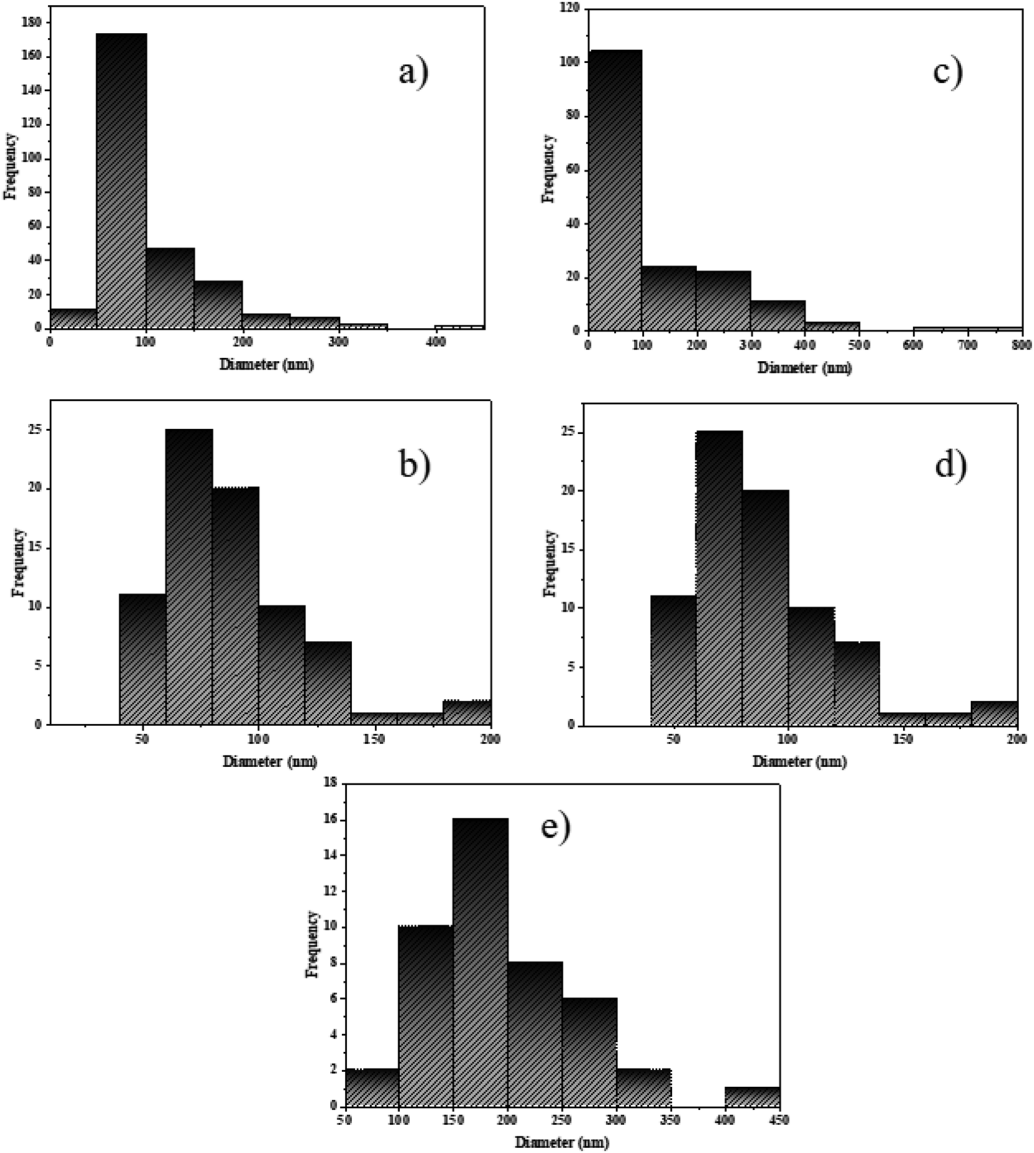
The particle size distribution histogram of NaY(WO_4_)_2_ samples prepared by (a) glucose, (b) fructose, (c) lactose, (d) cellulose, and (e) starch using Digimizer as the image analyser.

Fig. 3a shows that most particles are limited between 50 and 100 nm. Fig. 3b shows a particle size of approximately 75 nm. The particle size distribution for NaY(WO_4_)_2_ in Fig. 3c showed a narrow and individual distribution below 100 nm. Fig. 3d displays an average size of NaY(WO_4_)_2_ between 50 and 125 nm. Also, Fig. 3e shows that large particle frequency is more than the small particles, and the medium size of NaY(WO_4_)_2_ was approximately 197 nm.

**Fig. 4:**
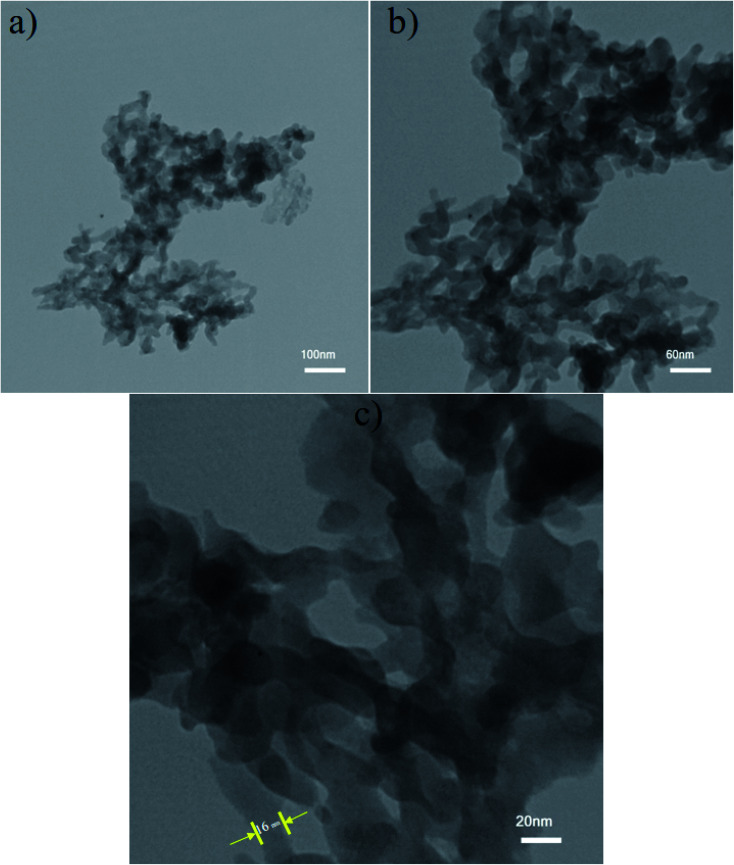
TEM images of NaY(WO_4_)_2_ at (a) 100 nm, (b) 60 nm, and (c) 20 nm scale bar fabricated by fructose as the optimum sample (S2). Particle shape of the NaY(WO_4_)_2_ is identified by TEM images.

The morphological properties of NaY(WO_4_)_2_ determined in optimized conditions (sample S2 synthesized using fructose as the capping agent) were examined using TEM images. The particle shape of the sample corroborated with the SEM image. The average size of the particles measured from TEM images using Digimizer software as the image analyzer was approximately 20 nm (Fig. 4a–c).

### 3.2 Structure and purity of NaY(WO_4_)_2_

**Fig. 5:**
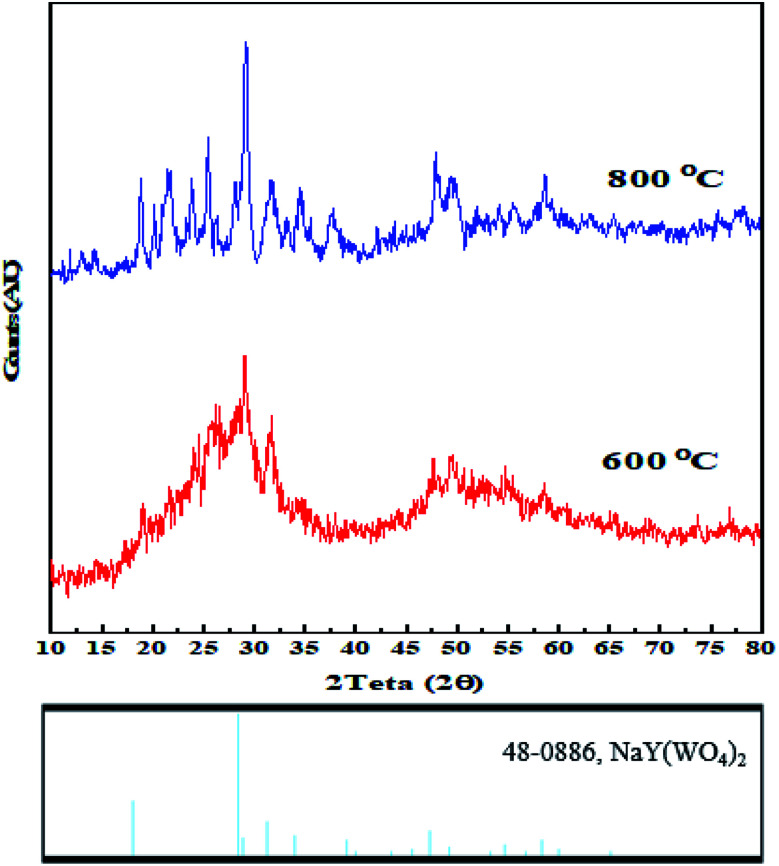
X-ray diffraction patterns of sodium yttrium tungstate nanoparticles fabricated by the hydrothermal method using fructose as the capping agents at 600 °C and 800 °C.

The as-synthesized samples were identified by various techniques. The primary way to nanostructure crystallite and phase identification is the X-ray diffraction pattern that is suitable and proper to check the samples' phase characterization. Fig. 5 indicates the XRD data of NaY(WO_4_)_2_ prepared *via* the hydrothermal method utilizing fructose (S2) at 600 and 800 °C. It is noticeable that the pattern of the fabricated sample S2 belongs to the sodium yttrium tungstate nanoparticle with tetragonal phase, showing that the sample is clear of unspecified impurity (JCPDS number 48-0886, space group number: 62, and space group: 141/*a*). When the temperature of calcination was increased from 600 °C to 800 °C, the size of the grain increased due to the high temperature heat treatment. The quantity of grain size was determined using the Scherrer equation from X'pert software (eqn (2)).^45^2
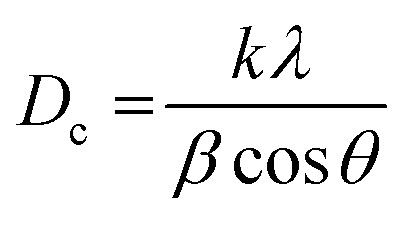
*β* is FWHM, *k* is the shape factor, which is about 0.9; *λ* is the source wavelength utilized in the XRD apparatus, and *θ* is the angle of each diffraction. The grain size of the as-prepared products was computed *via* calculation using the most intense peak in XRD patterns.^46^ Grain sizes at 600 °C and 800 °C were 20 nm and 29 nm, respectively.

**Fig. 6:**
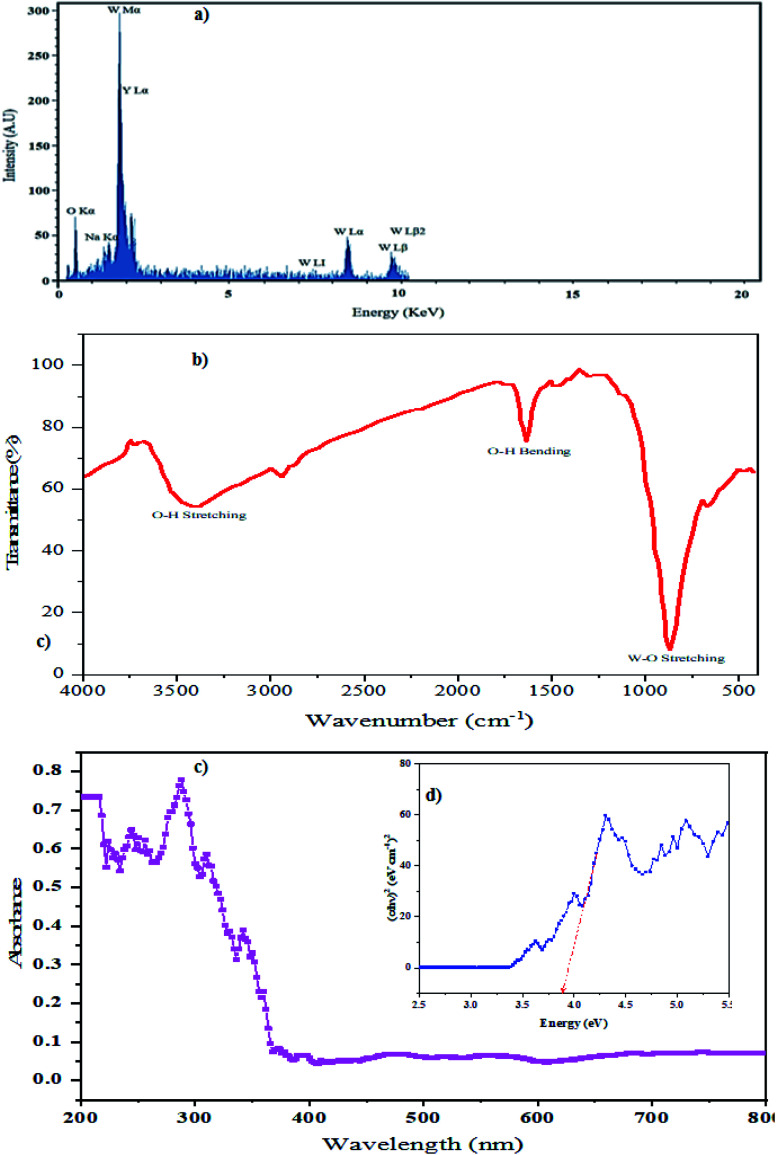
(a) EDS result of NaY(WO_4_)_2_ fabricated by fructose as the optimum sample S2, (b) FT-IR spectrum of the as-synthesized NaY(WO_4_)_2_ nanoparticles, (c) Drs spectrum of the sample S2, and (d) curve (*αhν*)2 against *hν* of sample S2.

According to the EDS pattern in Fig. 6a, only Na, Y, W, and O elements were identified. The atomic ratio of sodium : yttrium : tungsten was 1 : 1 : 2, indicating the excellent purity of the as-synthesized NaY(WO_4_)_2_ nanoparticles (sample S2). The atomic percentages of the elements were 0.56%, 6.821%, 12.03%, and 80% for Na, Y, W, and O, respectively.

Fig. 6b demonstrates the FT-IR spectrum of NaY(WO_4_)_2_ (sample S2). The narrow and sharp vibration mode at 850 cm^−1^ is attributed to W–O stretching. Also, the band about the 698–705 cm^−1^ region denotes the O–W–O bending vibrations. The W=O band is located at 980 cm^−1^ related to the stretching mode. Broad bands at 1619 cm^−1^ and also 3396 cm^−1^ are from surface absorbed water, which are attributed to H_2_O and O–H stretching vibration modes.^47^

### 3.3 Optical properties of NaY(WO_4_)_2_

Photodegradation performance is dependent on the morphology, bandgap quantity, and also the light-absorbing capacity of a photocatalyst. To gain the optical bandgap of NaY(WO_4_)_2_ formed in the sample obtained under optimized conditions (sample S2), a DRS absorption chart was obtained (Fig. 6). The bandgap quantity is calculated by the Tauc equation.^48^

The DRS spectrum of the fabricated photocatalyst is showed in Fig. 6c. The absorption band near 290 nm is related to the intensive NaY(WO_4_)_2_. Additional bands nearby 330 and 350 nm are assigned to the contaminants or vacant sites.^49^ The bandgap quantity of the as-prepared sample S2 can be computed based on the absorption spectrum using the Tauc equation by extrapolating the linear part of the Tauc diagram (Fig. 6d).^3,50^ The bandgap energy quantity of the as-prepared NaY(WO_4_)_2_ sample (sample S2) was found to be 3.85 eV. Therefore, these nanoparticles can perform as photocatalysts in dye degradation.

### 3.4 Photocatalytic activity

In this study, the photodegradation performance of NaY(WO_4_)_2_ was investigated by photo degradation of two dyes (as the simulators of water pollutants) under defined light irradiation. In addition, excessive photocatalysis coverage with absorbed dye molecules can exceptionally reduce photocatalytic efficiency. However, the samples were placed in dark media for 30 min before they were utilized for the photodegradation process. The influence of various parameters including morphology, type of dye, and influence of dye dosage on photocatalytic performance were examined.

**Fig. 7:**
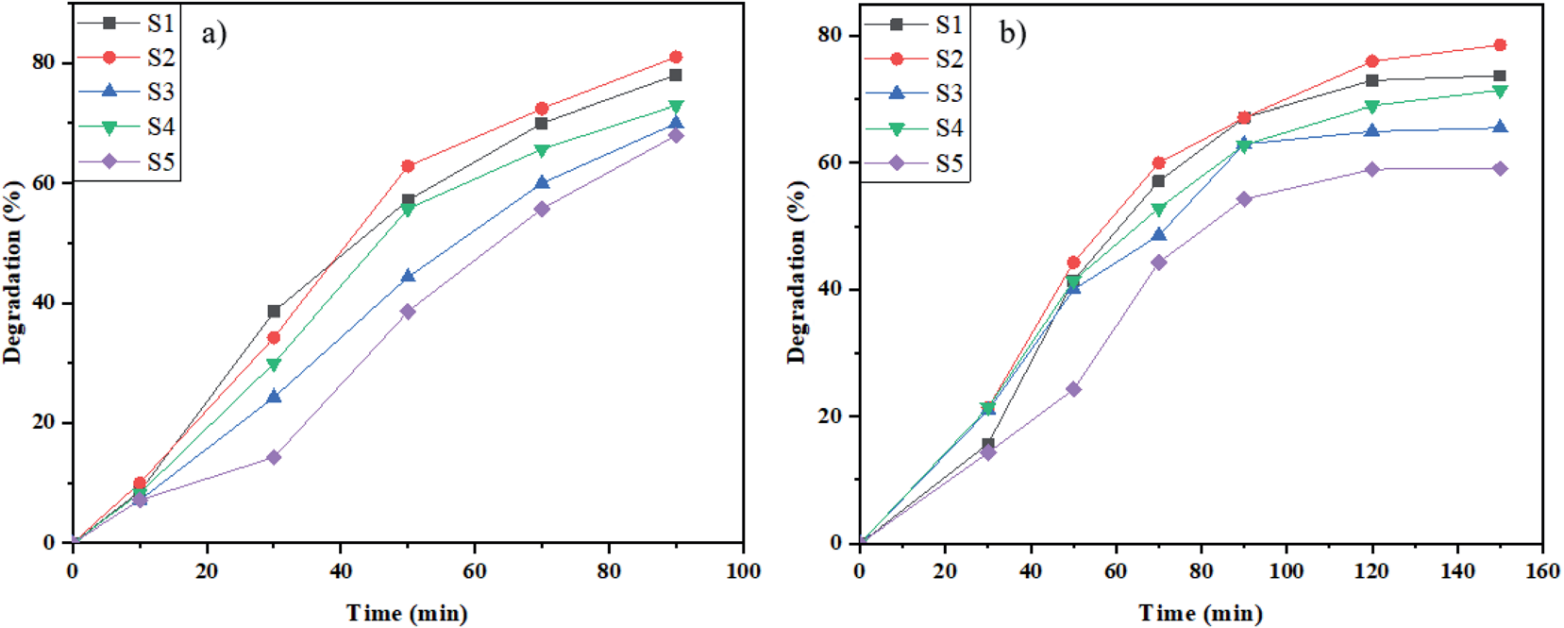
Photocatalytic elimination of M.O below the lighting of (a) UV, and (b) Vis on NaY(WO_4_)_2_ nanostructures (sample S1–S6).

As shown in Fig. 7a, the photodegradation of methyl orange (M.O) at the defined time was accomplished in the presence of UV and Vis irradiation. The UV photodegradation in 90 min was 78%, 81%, 70%, 73%, and 68% for S1, S2, S3, S4, and S5, respectively. In addition, the degradation percentage of M.O induced by Vis light was 73%, 78%, 65%, 71%, and 59% for S1, S2, S3, S4, and S5 samples, respectively (Fig. 7b). The highest degradation percentage in the presence of induced radiation was by as-prepared NaY(WO_4_)_2_ nanoparticles synthesized using fructose. The narrow size distribution and the proper bandgap caused high degradation of an azo dye in the presence of UV and Vis light.

**Fig. 8:**
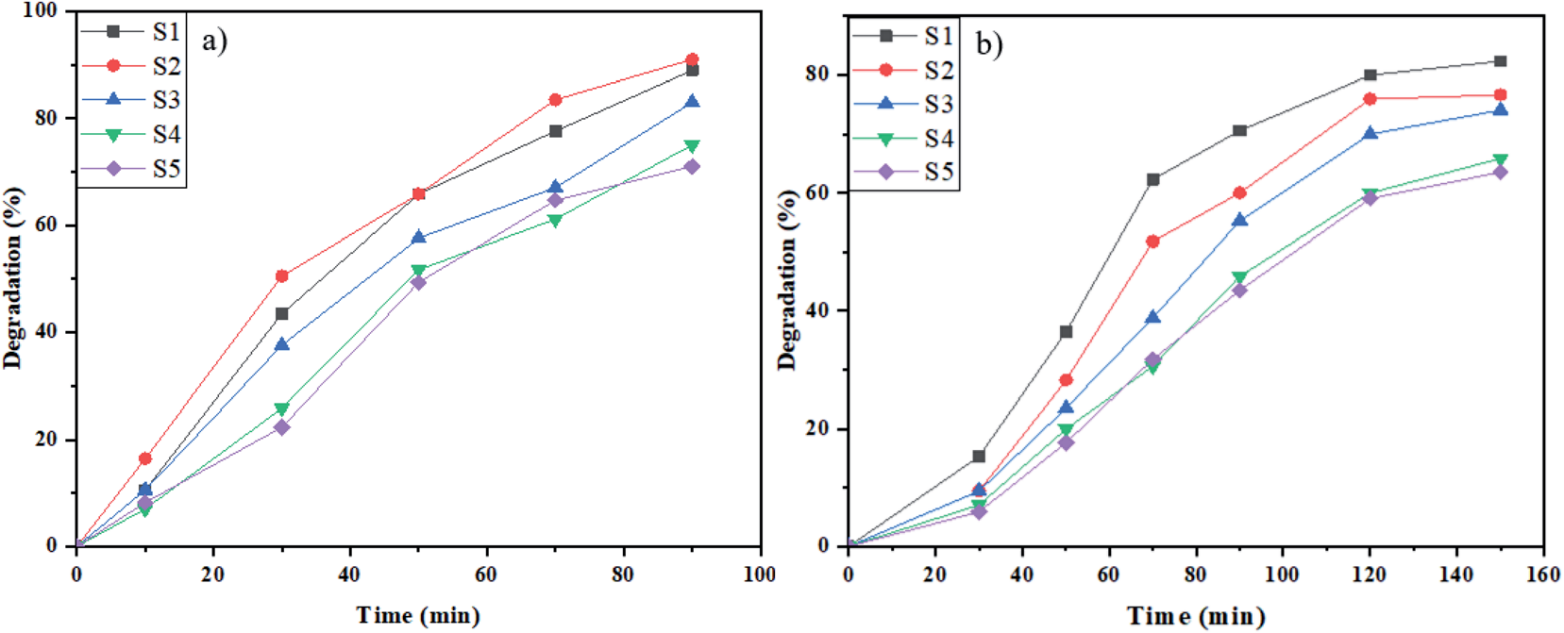
Photocatalytic elimination of Rd.B below the lighting of (a) UV, and (b) Vis on NaY(WO_4_)_2_ nanostructures (sample S1–S6).

Rhodamine B (Rd.B) is a typical textile organic dye that was degraded by samples S1–S5 in the proximity of the induced light at the determined period. As demonstrated in Fig. 8a, the percentage of degradation for samples S1–S5 in 90 min in the presence of UV radiation was 89%, 91%, 73%, 75%, and 71%, respectively. Furthermore, the photodegradation of Rd.B in 120 min in the presence of Vis radiation was 80% (sample S1), 76% (sample S2), 70% (sample S3), 60% (sample S4), and 59% (sample S5) (Fig. 8b). The samples prepared by glucose and fructose as the capping agents had the maximum percentage of degradation. The appropriate morphology and good size distribution of these samples increased the degradation percent of Rd.B induced by UV and Vis light irradiation.

**Fig. 9:**
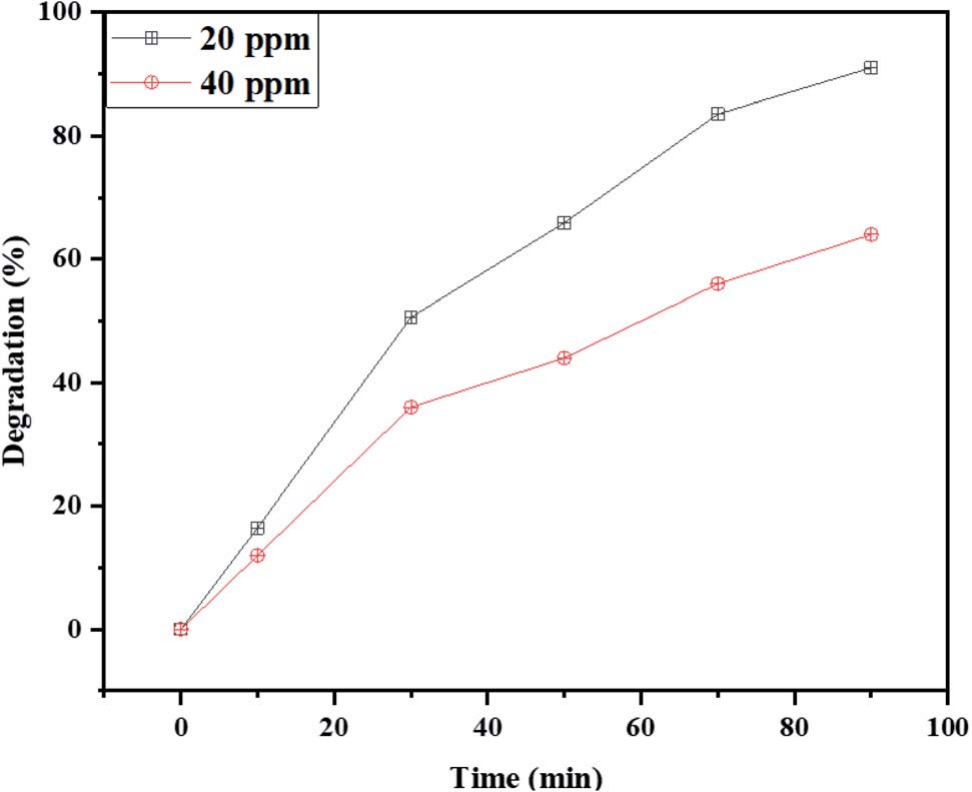
The effect of the Rd.B dosage on the photocatalytic activity of NaY(WO_4_)_2_ nanoparticles (sample S2).

Furthermore, the influence of dye dosage on the photodegradation process was also examined. Fig. 9 indicates the Rd.B concentration influence on the photodegradation efficiency of NaY(WO_4_)_2_ nanoparticles using fructose as the capping agent (sample S2). The results revealed that with the doubling of the concentration of Rd.B from 20 ppm to 40 ppm, the degradation of dye changed from 91% to 64%. This can be due to the excessive quantity of Rd.B in the reaction media, which increased the photocatalyst coverage and also decreasing the incident photon penetration in the suspension, consequently reducing the photodegradation efficiency of NaY(WO_4_)_2_.

The suggested reaction mechanism of dye degradation by NaY(WO_4_)_2_ nanostructures can be shown as:^51^NaY(WO_4_)_2_ nanoparticle + *hν* → NaY(WO_4_)_2_ nanoparticle* + electron (e^−^) + hole (h^+^)hole (h^+^) + H_2_O → OH˙ + H^+^2 hole (h^+^) + 2H_2_O → H_2_O_2_ + 2H^+^electron (e^−^) + O_2_ → O_2_^−^˙O_2_^−^˙ + 2OH˙ + H^+^ → H_2_O_2_ + O_2_H_2_O_2_ → 2OH˙O_2_^−^˙ + dye → photodegraded products

As shown in Fig. 7 and 8, the best performance in dye degradation was attributed to the sample S2 (synthesized by fructose). It is normally assumed that an increase in the rate of electron (e^−^)–hole (h^+^) recombination reduces the photodegradation efficiency.^52^ The sample S2's appropriate and monotonic bandgap and morphology, decreased chances of the e^−^–h^+^ recombination compares to other specimens.

**Fig. 10:**
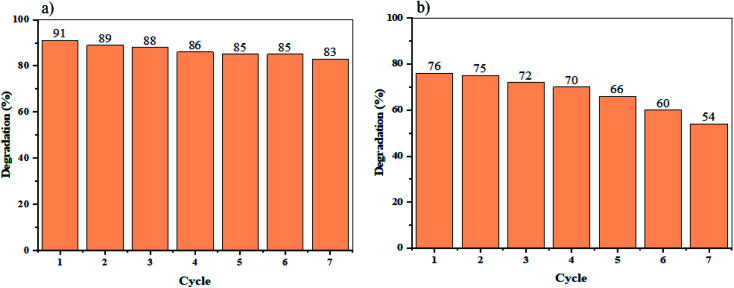
Recycling of the optimized sample (S2) for Rd.B elimination under (a) UV light, and (b) Vis light radiation.

To defining the reusability of NaY(WO_4_)_2_ nanoparticles as photocatalysts, the photodegradation process of sample S2 (the sample with the maximum percentage of degradation), was repeated and reused seven times. Consequently, the results indicate that the degradation of Rd.B under UV and Vis light irradiation declined slightly, after only four cycles of photoreaction, demonstrating superb stability and reusability of the as-synthesized sample (see Fig. 10).

## 4. Conclusion

In summary, NaY(WO_4_)_2_ nanoparticles have were prepared successfully by hydrothermal method-assisted heat treatment. Capping agents such as glucose, fructose, lactose, cellulose, and starch were utilized as morphology modifiers in the presence of Na_2_WO_4_ and Y(NO_3_)_3_ precursors to fabricate NaY(WO_4_)_2_ nanoparticles. Subsequently, the physical and chemical characteristics of as-synthesized NaY(WO_4_)_2_ nanostructures were analyzed by various techniques. By utilizing the SEM images, sample S2 was chosen as the optimized sample with a 78.5 nm average size. Also, the size distribution of the samples was analyzed by the Digimizer software. Extrapolating the Tauc plot, the bandgap of the sample S2 was calculated to be nearly 3.85 eV. Thereupon, the photocatalytic performance of the NaY(WO_4_)_2_ nanostructures was investigated by degrading M.O and Rd.B. The results indicated that the optimized sample S2, had 81% and 78% photodegradation of M.O, in the UV and Vis light regions, respectively. Also, the rate of photodegradation of Rd.B under UV and Vis radiation was 91% and 76%, respectively. Following on that, the effect of dye dosage revealed that an increase in the dye dosage caused loss in photocatalytic efficiency. Finally, the recycling and reusability of the photocatalyst was studied, by repeating the degradation of Rd.B under UV and Vis light seven times. After four-cycles, a slight reduction in the efficiency of photodegradation was observed.

## Conflicts of interest

The authors declare that there are no conflicts of interest regarding the publication of this manuscript.

## Supplementary Material
